# Lévy Versus Wiener: Assessing the Effects of Model Misspecification on Diffusion Model Parameters

**DOI:** 10.1007/s42113-025-00248-6

**Published:** 2025-05-27

**Authors:** Tuba Hato, Lukas Schumacher, Stefan T. Radev, Andreas Voss

**Affiliations:** 1 Institute of Psychology, Heidelberg University, Hauptstrasse 47-51, 69117 Heidelberg, Germany; 2 Institute of Psychology, University of Basel, Missionsstrasse 60/62, 4055 Basel, Switzerland; 3 Center for Modeling, Simulation and Imaging for Medicine, Rensselaer Polytechnic Institute, 110 8th St, 12180 Troy, NY USA

**Keywords:** Lévy-flight model, Diffusion decision model, Parameter recovery, Model misspecification, Amortized Bayesian inference

## Abstract

The Lévy-flight model (LFM; Voss et al., *Psychonomic Bulletin & Review, 26*, 813–832, 2019) modifies the diffusion decision model (DDM; Ratcliff, *Psychological Review, 85*(2), 59, 1978) by integrating a heavy-tailed noise distribution into evidence accumulation. Initial studies suggest that the LFM can competitively fit human response time distributions. Consequently, they argued that the LFM may reflect the binary decision process more faithfully. If this is the case, analyzing data with a classical DDM may misrepresent the underlying latent processes. In the present study, we explore the estimation biases that arise when data conforming to an LFM are analyzed with the DDM. We conducted extensive simulations using both basic and full versions of the DDM and the LFM, employing cross-fitting through simulation-based inference with neural networks as implemented in the BayesFlow framework. Given the susceptibility of neural networks to misspecification, we additionally employ a Markov chain Monte Carlo (MCMC) approach as a benchmark for the neural estimates. Our results demonstrate that neural networks and MCMC exhibited nearly identical estimation performance for the basic DDM. Importantly, the basic DDM showed notable overestimation of boundary separation and underestimation of non-decision time when fitted to data generated by the LFM. The full DDM significantly overestimated inter-trial variabilities in starting point and non-decision time, while still overestimating boundary separation. Subsequently, we applied the models to experimental data from a task incorporating speed and accuracy manipulations. Both models reflected the expected effects of the experimental manipulation on boundary separation and non-decision time, additionally, the stability parameter $\alpha$ differed between conditions in the LFM. In conclusion, while our simulations indicate that DDM-based analyses may introduce biases when the true data-generating process aligns more closely with the LFM, applying both models to experimental data led to convergent interpretations.

## Lévy Versus Wiener: Assessing the Effects of Model Misspecification on Diffusion Model Parameters

A popular modeling approach in decision-making assumes that (rapid) binary decisions can be represented as a sequential sampling process where the decision-maker accumulates evidence until the process crosses a boundary. The corresponding evidence accumulation models (EAMs) have gained popularity due to their ability to decompose response times (RTs) into latent psychological processes (Evans & Wagenmakers, 2019). Among these models, the Diffusion Decision Model (DDM) is the most prominent and extensively studied (Ratcliff, 1978; Ratcliff & McKoon, 2008; Voss et al., 2013). The DDM formalizes evidence accumulation as a Wiener process with linear drift and Gaussian noise that evolves between two decision boundaries. Each decision boundary maps onto a categorical choice between the two alternatives, and the process is terminated as soon as the accumulated evidence reaches one of the boundaries.

Since the introduction of the DDM in psychology (Laming, 1968; Ratcliff, 1978; Stone, 1960), the model has been successfully applied in various domains of cognitive psychology, such as recognition memory (Ratcliff et al., 2004), lexical (Wagenmakers et al., 2008), and perceptual decision-making (Ratcliff et al., 2003, 2006; Voss et al., 2004a). Over the last decades, several modifications to the original model formulation have been introduced to address limitations, such as its inability to account for fast or slow errors. The proposed modifications include the incorporation of inter-trial variability parameters (Ratcliff & Rouder, 1998; Ratcliff & Tuerlinckx, 2002) or collapsing boundaries (Voskuilen et al., 2016; Bowman et al., 2012; Voss et al., 2019). The DDM, along with its extended versions, has become the default model for studying binary decision-making processes.

The Lévy-flight model (LFM; Voss et al., 2019) modifies the DDM by replacing the Gaussian noise distribution of the Wiener diffusion process with a heavy-tailed distribution. Specifically, noise in evidence accumulation is modeled by the Lévy alpha-stable distribution, where the additional parameter $\alpha$ determines the heaviness of the tails. This changes the properties of the model fundamentally, as a heavy-tailed noise distribution allows for sudden “jumps” in evidence accumulation, thereby changing the model’s predictions for RTs and errors. Specifically—in contrast to the basic DDM—the LFM does not predict identical mean RTs for correct and error responses (cf. Link and Heath, 1975). Thereby, the LFM offers an alternative explanation for fast errors that are often observed in perceptual decision-making (e.g., Laming, 1968). In contrast, the DDM accounts for fast errors by incorporating inter-trial variability parameter of the starting point, which can lead to poor parameter recovery (Voss et al., 2019). In particular, the family of alpha-stable distributions also includes the normal distribution (for $\alpha =2.0$), implying that the DDM is a special case of the more general LFM. This characteristic enables the LFM to capture a broader range of behavioral patterns (Wieschen et al., 2020), particularly those observed under time-pressure (Voss et al., 2019; Rasanan et al., 2024), compared to the basic DDM. Therefore, previous work (Voss et al., 2019; Wieschen et al., 2020, 2023) argued that the LFM may reflect the binary decision process more faithfully, implying that fitting the basic DDM as a default model may result in model misspecification. Given these differences, it is important to consider the potential implications of using the DDM when the underlying data may be better represented by the LFM.

In cognitive modeling, parameter estimation is critical to interpret latent processes; however, model misspecification can introduce biases, leading to incorrect interpretations. Building on previous research, our main question is what biases emerge in parameter estimates when the true data-generating process aligns more closely to the LFM, but the DDM is used for data analysis. In other words, we examine the consequences of misspecifying the LFM as the DDM. To address this issue, we conducted a large-scale simulation study, where the DDM was fitted to LFM-generated data. We then estimated parameters using the misspecified DDM and demonstrated the resulting biases *in silico*. Additionally, we fitted our models to real data from Experiment 1 of the study by Evans et al. (2017). The experiment entailed a perceptual decision task in which participants were instructed to respond as fast as possible in some blocks and as accurate as possible in other (for more details, see Evans et al., 2017).

### Model Details

The basic DDM comprises four key parameters: boundary separation (*a*), drift rate (*v*), starting point (*z*), and non-decision time ($t_0$). Boundary separation represents the amount of evidence required for an individual to make a decision. When a participant adopts a more conservative decision strategy, their boundary separation becomes larger, leading to slower yet more accurate responses (Ratcliff & Rouder, 1998; Voss et al., 2004b). Conversely, narrow boundary separations are adopted by less cautious decision-makers (Evans, 2021) and will lead to faster responses and lower accuracy (Starns & Ratcliff, 2010; Evans, 2021). The drift rate indicates the speed and direction of evidence accumulation. With a higher (lower) absolute drift rate, the decision process will—on average—reach a boundary faster (slower). Generally, easier tasks lead to higher drift rates (Ratcliff, 2014; Voss et al., 2004b), and drift has also been found to correlate with intelligence (Lerche et al., 2020; Schmiedek et al., 2007). In unbiased decisions, the starting point is centered between the two boundaries. Otherwise, the starting point might be closer to one boundary, leading to faster RTs for the biased decision. Finally, the non-decision time accounts for the duration of all processes unrelated to evidence accumulation, such as stimulus encoding and response execution.

Often, for a full DDM analysis, additional inter-trial variability parameters are included, which makes the model much more flexible to account for different data patterns. For instance, when speed is prioritized or the task is simple, errors are usually faster than correct responses (Wagenmakers et al., 2008; Ratcliff & Rouder, 1998). The assumption of trial-to-trial variability of the starting point ($s_z$; Ratcliff and Rouder, 1998; Smith et al., 2014) allows the model to account for fast errors. This parameter represents fluctuations in expectations about the upcoming stimuli. Drift rate variability ($s_v$) can explain slow errors (Ratcliff & Rouder, 1998). The non-decision time variability parameter ($s_{t_0}$) was introduced to account for very fast responses (Ratcliff & Tuerlinckx, 2002).

The LFM includes the same parameters as the DDM, with the additional stability parameter $\alpha$, which governs the shape of the noise distribution.^1^ Theoretically, $\alpha$ can range from 0 to 2, but in the context of the LFM, it is typically observed within the interval [1, 2] (Voss et al., 2019). When $\alpha$ approaches 2.0, the noise distribution becomes normal, and the LFM converges to the DDM. Lower values of $\alpha$ increase the likelihood of extreme random events in evidence accumulation, also known as “jumps.” The LFM can account for fast errors without the additional assumption of inter-trial variability of the starting point. Nevertheless, also for the LFM, all inter-trial variability parameters can be included.

If $\alpha$ is small, evidence accumulation is strongly influenced by jumps. Consequently, in some trials, the decision process might jump at very early stages of evidence accumulation directly to a random boundary, thus leading to fast errors. Since the DDM does not allow for jumps in evidence accumulation, data that incorporates fast random responses will probably lead to a biased parameter estimation if the DDM is applied: For example, a substantial number of fast random responses might be explained in the DDM framework by a small boundary separation in combination with a low drift rate.

One notable disadvantage of the LFM is the lack of a closed-form likelihood function for predicted RTs. This makes traditional likelihood-dependent inference methods—such as Markov chain Monte Carlo (MCMC)—inapplicable. As a result, a likelihood-free approach is required to fit the LFM to the data. For this purpose, we employ amortized Bayesian inference with neural networks (Radev et al., 2023).

### Previous Findings

In recent studies, comparing the goodness-of-fit of the LFM and the DDM for binary decision data, the LFM outperformed the DDM (Voss et al., 2019; Wieschen et al., 2020). Voss et al. (2019) fitted the model to the data from an (easy) single stimulus and a (more difficult) multi-stimulus task. The authors report that $\alpha$ depends on the task: While for the difficult task, evidence accumulation resembled a Wiener process (mean $\alpha \approx 1.8$), stability of evidence accumulation was reduced for the single stimulus task ($\alpha \approx 1.5$; for details, see Voss et al., 2019). Thus, results indicate that deviations from predictions of the basic DDM are notably stronger for the easy and faster task.

Wieschen et al. (2020) subsequently replicated this finding in a larger study with more observations per participant. Both basic and full versions of the DDM and LFM were fitted to the data. The basic model included two drift rates ($v_0$, $v_1$), a boundary separation (*a*), a starting point (*z*), and a non-decision time ($t_0$). The full models additionally included all inter-trial variability parameters ($s_v$, $s_z$, $s_{t_0}$). Again, the LFMs showed a better goodness-of-fit compared to the corresponding DDMs, with $\alpha$ values around 1.6.

In a subsequent study, age effects were examined by comparing the performance of young and older adults on a letter-number discrimination task using the LFM (Wieschen et al., 2023). In diffusion modeling, age effects are typically associated with older individuals adopting more conservative strategies, characterized by larger boundary separations and longer non-decision times (Ratcliff et al., 2003, 2004; Thapar et al., 2003; Ratcliff & McKoon, 2023; Theisen et al., 2021). In this study, additionally, the stability of evidence accumulation $\alpha$ was significantly lower for younger participants (1.6) than for older participants (2.0). The authors concluded that the behavior of younger participants can be better described by the LFM, whereas older participants’ performance is described adequately by the DDM.

The DDM and the LFM are both based on a similar theoretical framework, but the models differ fundamentally in their characterization of the decision-making process. The findings summarized above suggest that the LFM provides a better fit, particularly for data from younger participants and easier tasks. However, numerous studies have applied the DDM to data collected from young participants, involving easy tasks and time-pressured conditions. In the present paper, we hypothesize that in many previous studies employing the DDM, the true decision process is better reflected by a Lévy Flight (with $\alpha \ll 2.0$). As such differences are not accounted for in DDM analyses, results might be biased. Problematically, the DDM was often recommended as a model for fast perceptual decisions (e.g., Ratcliff and McKoon, 2008), where problems seem to be the largest. This is also evident from more recent studies suggesting the goodness-of-fit of the DDM is better for data from more complex tasks with longer RTs than for simple perceptual decisions (Lerche et al., 2020).

This potential issue is particularly problematic if $\alpha$ differs between experimental conditions or groups. For example, the findings of Wieschen et al. (2023) suggest that DDM parameters in cognitive aging studies may exhibit stronger biases for younger participants, where the DDM may offer a better fit, while showing little to no bias for older participants. A similar argument can be made for speed-accuracy studies. In both cases, observed differences between groups or conditions in the behavioral data may, in part, reflect differences in the stability parameter $\alpha$. Since the DDM cannot account for this effect, such differences may be mapped by other parameters, leading to potentially biased estimates. A related issue was investigated by Donkin et al. (2011) in a simulation study comparing how the Linear Ballistic Accumulation model (LBA) and DDM parameters map onto each other. Although they observed mismappings in the simulation study, the models reached the same conclusions when fitted to experimental data (a similar finding was reported by Forstmann et al. (2008)). Therefore, it is essential to investigate these claims both theoretically, through simulation studies, and empirically, by applying the models to experimental data.

### Contributions

To address the above issues of model misspecification, we conducted an extensive simulation study to quantify the extent of bias in parameter estimates as the true data-generating model diverges from the DDM. We demonstrate the DDM’s fit in well-specified scenarios using both Markov chain Monte Carlo (MCMC) as implemented in Stan (Carpenter et al., 2017) and neural networks as implemented in BayesFlow (Radev et al., 2023). More importantly, we apply cross-fitting to investigate the impact of model misspecification. Our results suggest that when data generated by the basic LFM is analyzed with the basic DDM, boundary separation (*a*) is overestimated and non-decision time ($t_0$) is underestimated. Additionally, when the full DDM is misspecified, we observe overestimation of the inter-trial variability for starting point ($s_z$) and non-decision time ($s_{t_0}$) along with an underestimation of the boundary separation (*a*) as a function of misspecification severity. We also demonstrate that neural networks perform well under model misspecification, offering robust estimates. To our knowledge, this is the first study to systematically identify and quantify biases introduced by model misspecification between the DDM and the LFMs. The full code for this study is available on GitHub (https://github.com/HatoTuba/LFM_model-misspecification.git).

**Fig. 1:**
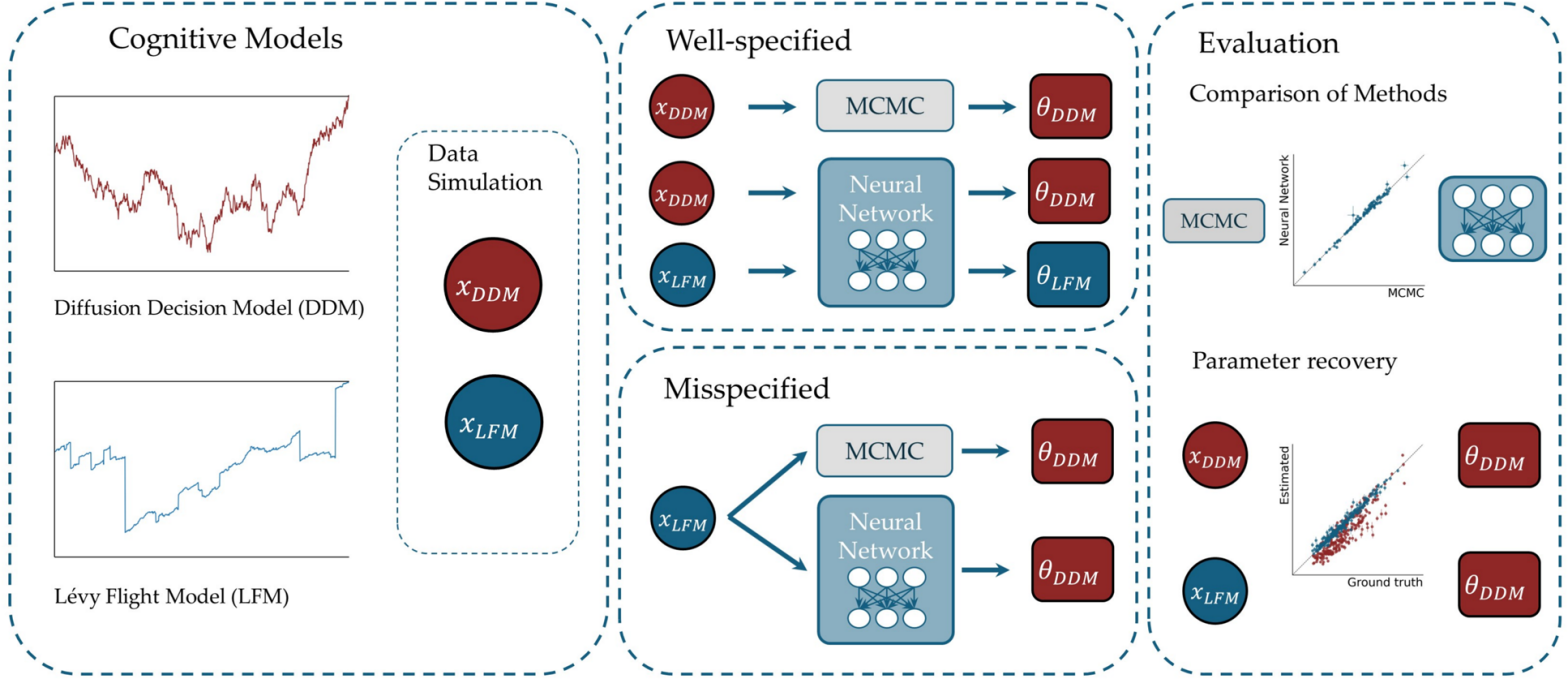
Workflow of the present study. Data were simulated independently using either the basic or full versions of the DDM or the LFM. Parameter estimation was performed using neural networks and, for the basic diffusion model, also via MCMC to obtain a benchmark for parameter recovery. The well-specified conditions were simulated to evaluate computational faithfulness. Cross-fitting was used to assess the impact of model misspecification on parameter estimates

## Method

### Model Formulation

The general evidence accumulation process for the DDM is defined in Eq. 1, where *dx* denotes the accumulated evidence, *v* denotes the rate of information accumulation (drift rate), and $\xi$ denotes a stochastic component (i.e., the noise). In the DDM, noise follows a standard-normal distribution. Our basic version of the DDM incorporates two different drift rates targeting each boundary, representing two different stimulus types, totaling five parameters ($v_1$, $v_2$, *a*, *z*, $t_0$). Additionally, the full DDM encompasses inter-trial variability parameters for drift rate ($s_v$), starting point ($s_z$), and non-decision time ($s_{t_0}$). Drift rates were implemented with a mean of *v* and a standard deviation of $s_v$. Variability in the starting point and non-decision time were modeled as uniform distributions, with ranges defined by $s_z$ and $s_{t_0}$, and centered around *z* and $t_0$.

1 $$\begin{aligned} dx = v \, dt + \sigma \, d\xi \quad \text {with} \quad \xi \sim \text {Normal}(0, 1) \end{aligned}$$

In the LFM, the noise within the accumulation process follows a heavy-tailed $\alpha$-stable distribution Eq. 2.

2 $$\begin{aligned} \xi \sim \text {AlphaStable}(\alpha , \mu = 0, \sigma = \frac{1}{\sqrt{2}}, \beta = 0) \end{aligned}$$

The parameters $\alpha$, $\mu$, and $\beta$ characterize the $\alpha$-stable distribution, where $\alpha \in (0, 2]$ is known as the stability index (Lévy index). The $\alpha$ parameter is constrained to the interval [1, 2] in our implementation as this range covers observed values from previous LFM studies. Given that empirical studies report mean posterior $\alpha$ values of approximately 1.6 (Wieschen et al., 2020, 2023), we opted for a broader interval. Similar to our implementation of the DDM, the LFM incorporates two drift rates, totaling six parameters ($v_1$, $v_2$, *a*, *z*, $t_0$, $\alpha$). Additionally, the full LFM incorporates all inter-trial variability parameters ($s_v$, $s_z$, $s_{t_0}$), resulting in a total of nine parameters.

### Simulation-Based Inference

Simulation-based inference (SBI; Cranmer et al., 2020) offers a powerful framework for estimating complex models with arbitrary likelihoods. SBI relies solely on model simulations to recover parameters without explicit likelihood evaluations. SBI approaches commonly employ generative neural networks to perform fully Bayesian inference (i.e., to obtain the full posterior distribution over model parameters). For the present study, we set up our simulation-based workflows using the BayesFlow Python library (Radev et al., 2023).

Our SBI setup entails the following steps. First, we formulated generative models for each version of the DDM and LFM. The models take selected parameters from $\theta _{all} = (v_1, v_2, s_v, a, z, s_z, t_0, s_{t_0}, \alpha )$ as inputs and generate a sample of RTs and choices as outputs. Second, we simulated a large number of datasets (100,000 per model) using these generative models, with parameters randomly drawn from prior distributions (Table  4). Third, we trained the generative networks for 100 epochs until convergence. Following training, the pre-trained network can be used for any number of new datasets.

Crucially, the performance of neural posterior estimators can degrade when the simulation program fails to accurately represent real-world conditions at test time, making these methods susceptible to model misspecification (Schmitt et al., 2023). In contrast, MCMC, here implemented in Stan (Carpenter et al., 2017), is generally more robust to model misspecification; thus, it serves as a gold-standard benchmark for our neural network method. Notably, to our knowledge this work is the first to explore model misspecification using SBI with both the DDM and the LFMs.

### Model Misspecification Setting

The workflow of the study is illustrated in Fig. 1; for training and inference, we randomly drew parameter sets from prior distributions designed to reflect typical values observed in empirical research. For evaluation, we generated four datasets, each consisting of 500 simulated trials of 1000 simulated participants corresponding to four different models: basic versus full models and DDM versus LFM. Data from all models and all 1000 simulated participants were fed to the neural networks, while MCMC was used to fit the DDM to 100 simulated participants from the basic DDM and LFM. In the basic models, inter-trial parameters were fixed at zero, while in the DDMs, the stability parameter $\alpha$ was set to 2.0. All other parameters were kept consistent within each parameter set across simulations. To represent two distinct stimulus types, we used separate drift rates, $v_1$ and $v_2$, each applied to half of the trials in every dataset.

### Evaluation Metrics

We calculate evaluation metrics in order to quantify the estimation bias in the misspecified scenario, and parameter recovery in both well and misspecified scenarios. Quantifying this bias provides a clear assessment of both the direction and magnitude of the estimation bias. Additional metrics—here, coefficient of determination ($R^2$) and posterior contraction (PC)—offer a more comprehensive evaluation of the parameter recovery performance. We assess these metrics using $N = 1000$ test simulations that were not included in the training phase.

**Table 1:** Parameter recovery for well-specified models

	Models	$v_1$	$v_2$	*a*	*z*	$t_0$	$s_v$	$s_z$	$s_{t_0}$	$\alpha$
$R^2$ values	Basic DDM (MCMC)	0.96	0.94	0.99	0.99	0.99				
	Basic DDM	0.93	0.93	0.99	0.99	0.99				
	Full DDM	0.88	0.87	0.99	0.98	0.99	0.34	0.07	0.90	
	Basic LFM	0.94	0.93	0.98	0.97	1.00				0.89
	Full LFM	0.88	0.89	0.96	0.96	0.98	0.63	0.08	0.80	0.69
PC values	Basic DDM (MCMC)	0.94	0.94	1.00	0.99	1.00				
	Basic DDM	0.94	0.94	1.00	0.99	1.00				
	Full DDM	0.89	0.89	0.99	0.98	0.99	0.49	0.17	0.94	
	Basic LFM	0.95	0.95	0.99	0.97	1.00				0.88
	Full LFM	0.91	0.91	0.99	0.96	0.99	0.75	0.15	0.85	0.73

Notes: *DDM*, diffusion decision model; *LFM*, Lévy-flight model. Parameters were recovered by an MCMC approach (Stan; in the first row) and by a neural estimation approach (BayesFlow; all other rows)

We define estimation bias as the average deviation of each estimated parameter value from the ground-truth simulated values:

3 $$\begin{aligned} \text {Bias}(\hat{\theta }, \theta ) = \frac{1}{N}\sum _{n=1}^N\frac{\hat{\theta }_n - \theta _n}{\sigma _{\theta }}, \end{aligned}$$

where $\hat{\theta }$ refers to a summary of the posterior (e.g., the posterior mean or median) and $\sigma _{\theta }$ denotes the prior standard deviation. We also aim to quantify estimation accuracy and uncertainty reduction in both well-specified and misspecified cases. Specifically, the posterior median was used as the summary statistic to assess the deviation from ground-truth simulated values. Appendix B presents simulation-based calibration (Talts et al., 2018) results as a more comprehensive evaluation of posterior credibility intervals. We quantify point estimation accuracy using the (predictive) coefficient of determination ($R^2$), which indicates how well the point estimates (posterior medians) align with the actual ground-truth parameter values:

4 $$\begin{aligned} R^2(\hat{\theta }, \theta ) = 1 - \frac{\sum _{n=1}^{N} (\theta _n - \hat{\theta }_n)^2}{\sum _{n=1}^{N} (\theta _n - \bar{\theta })^2}, \end{aligned}$$

Finally, posterior contraction (PC) measures the reduction of uncertainty elicited by updating a parameter’s prior distribution to the posterior,

5 $$\begin{aligned} \text {PC}(\theta , x) = 1 - \frac{\sigma ^2_{\theta \mid x}}{\sigma ^2_{\theta }}, \end{aligned}$$

where $\sigma ^2_{\theta \mid x}$ denotes the posterior variance and $\sigma ^2_{\theta }$ denotes the prior variance. The evaluation metrics were set to quantify the estimation bias in misspecified scenarios while assessing the accuracy and robustness of parameter recovery across both well-specified and misspecified cases. Our study uses the $\alpha$ parameters of the LFM as a proxy for misspecification severity, wherein lower values signify greater deviation from the basic DDM.

## Results

### Well-Specified Models

In this section, we describe the parameter recovery for the well-specified models, where models for parameter estimation make no false assumptions. These simulations serve as a baseline that gives a reference for the effects of model misspecification. Table 1 lists the quality of parameter recovery using $R^2$ and *PC* values for the well-specified models based on 1000 test sets per model. As demonstrated in the first-row of Table 1, MCMC and neural estimation demonstrated nearly identical performance, for the basic DDM. The drift rates ($v_1$, $v_2$) were consistently well-recovered across all models. Boundary separation was well-recovered for both the basic DDM, $R^2(a) =.99$, and for the basic LFM, $R^2(a) =.98$. PC values show that Bayesian information gain is quite high for the well-specified basic models.

Estimation quality decreased for the full models, particularly evident in the inter-trial variability parameters, which is typical for diffusion modeling (Lerche & Voss, 2016). The starting point variability parameter showed very poor parameter recovery, full DDM, $R^2(s_z) =.07$, and full LFM, $R^2(s_z) =.08$. Results for inter-trial variability for drift rate, full DDM, $R^2(s_v) =.34$, and full LFM, $R^2(s_v) =.63$, can be considered satisfactory. The variability for non-decision time demonstrated good performance for the full DDM, $R^2(s_{t_0} =.9$, and for the full LFM, $R^2(s_{t_0}) =.8$. Lastly, the $\alpha$ parameter exhibited decent recovery overall, poorer performance noted for the full LFM, $R^2(\alpha ) =.69$ compared to the basic LFM, $R^2(\alpha ) =.89$. Poor PC coincides with the poor parameter recovery of inter-trial variability parameters in both models (Table 1).

**Table 2:** Parameter recovery for misspecified models

	Data	Models	$v_1$	$v_2$	*a*	*z*	$t_0$	$s_v$	$s_z$	$s_{t_0}$
$R^2$ values	Basic DDM	Basic LFM	0.92	0.91	0.97	0.98	0.98			
	Full DDM	Full LFM	0.87	0.85	0.96	0.97	0.98	0.37	$-$0.02	0.85
	Basic LFM	Basic DDM (MCMC)	0.73	0.66	0.65	0.71	0.87			
	Basic LFM	Basic DDM	0.74	0.71	0.77	0.75	0.85			
	Full LFM	Full DDM	0.77	0.76	0.82	0.85	0.94	0.40	$-$1.17	0.29
PC values	Basic DDM	Basic LFM	00.93	0.93	1.00	0.98	1.00			
	Full DDM	Full LFM	0.89	0.89	0.99	0.98	0.99	0.50	0.04	0.93
	Basic LFM	Basic DDM (MCMC)	0.95	0.95	1.00	0.99	1.00			
	Basic LFM	Basic DDM	0.95	0.95	1.00	0.99	1.00			
	Full LFM	Full DDM	0.91	0.91	0.99	0.98	0.98	0.69	$-$0.13	0.90

Notes: *DDM*, diffusion decision model; *LFM*, Lévy-flight model. Parameters were recovered by an MCMC approach (Stan; third rows) and by a neural estimation approach (BayesFlow; all other rows). Because models are cross-fitted, the recovery of $\alpha$ parameter cannot be assessed

**Fig. 2:**
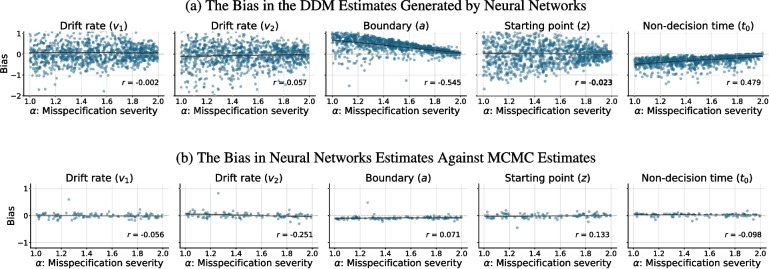
The bias plot depicts the quantification of bias as a function of misspecification severity when the basic DDM is fitted to data generated by the basic LFM. Smaller $\alpha$ values represent more severe deviation from the DDM, resulting in stronger bias. Depicted *r* values indicate the correlation between bias and $\alpha$ values. **a** The bias in the estimated parameters of the DDM across $\alpha$ values using neural estimation. **b** The bias in the basic DDM estimates obtained via neural networks, implemented with BayesFlow, to the gold-standard benchmark, MCMC, implemented with Stan

### Misspecified Basic Models

The impact of model misspecification was assessed by cross-fitting the models, where the DDM was fitted to LFM-generated data and *vice versa*. Here, we are interested in potential biases when analyzing LFM-generated data using the DDM. As the DDM is a special case of the LFM, fitting the LFM to DDM-generated data does not result in a model misspecification in a strict sense. Table 2 summarizes the metrics on parameter recovery. As expected, fitting the LFM to DDM-generated data is relatively unproblematic, as indicated by the fit indices which are in a similar range as for the well-specified models. However, $R^2$ values are notably lower, when LFM-generated data are analyzed using the DDM. The same pattern was observed in MCMC analyses as well. Note that the number of datasets analyzed with MCMC is much smaller (100 simulated participants using the basic models) compared to those analyzed with neural networks (1000 simulated participants using all models), as the former approach is computationally much more expensive.

Figure 2 illustrates the quantification of bias as a function of $\alpha$. Lower values of $\alpha$ denote a greater violation of the DDM’s assumption. Therefore, we expected stronger biases. Panel (a) shows the bias of DDM estimates when fitted to LFM-generated data using neural networks; panel (b) depicts the bias in neural network estimates compared to the MCMC estimates. For the neural network analyses (panel a), results indicate no bias in absolute drift rates and starting points. However, stronger effects of model misspecification are evident for boundary separation and non-decision time, where boundary is notably overestimated, non-decision time is underestimated. In the present simulations, we found the same pattern in the MCMC analyses (see Fig. 8).

### Misspecified Full Models

**Fig. 3:**
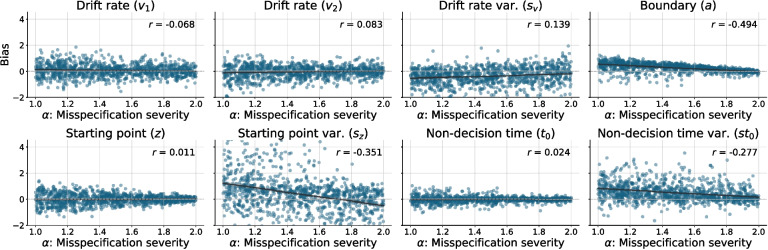
The bias plot depicts the quantification of bias in parameter estimation within the full DDM when it is fitted to data generated by the full LFM. Depicted *r* values indicate the correlation between bias and $\alpha$ values. Smaller $\alpha$ values represent more severe deviation from the DDM, leading to stronger bias. The bias is notably more pronounced in the inter-trial parameters

**Fig. 8:**
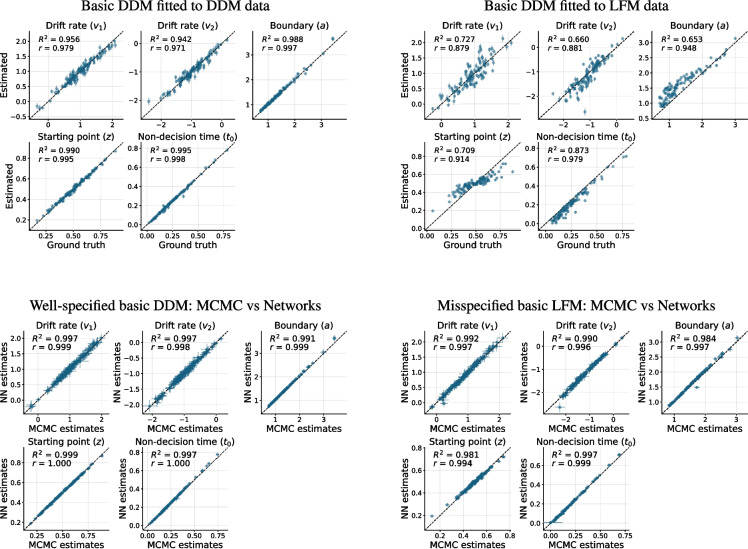
Parameter recovery plots for MCMC fits and comparison of estimates. The top section shows the parameter recovery plots for MCMC fits, while the bottom section compares MCMC and neural estimation estimates

The impact of model misspecification in the full models was also evaluated through cross-fitting, where the full DDM was fitted via neural networks to data generated by the full LFM. Fit metrics are presented in Table 2, and Fig. 3 depicts the quantified bias. Including inter-trial variability parameters gives the DDM a lot more flexibility to capture different patterns of data, including fast errors. Therefore, the estimation of the model’s core parameters (i.e., *a*, *z*, *v*, and $t_0$) are more robust here.

$R^2$ values indicate a similar recovery of core parameters as in the basic model, although *PC* is slightly worse. The inter-trial variability of the starting point ($s_z$) and non-decision time ($s_{t0}$) could not be recovered at all. Results regarding bias in estimates (Fig. 3) show that the DDM compensates for low $\alpha$ values with a large inter-trial variability of the starting point ($s_z$). This adjustment mitigates the biases observed in the basic model to some extent, as the bias in non-decision time is reduced almost to zero. However, for boundary separation (*a*), a notable overestimation of true values persists in the full DDM.

## Experimental Data Application

Following our simulation study, Experiment 1 of the study by Evans et al. (2017) was selected for the application of experimental data because it has similar characteristics as our simulation. The experiment was a perceptual discrimination task with two categories of stimuli, each corresponding to a separate drift rate, as in our simulations. Participants completed the task by choosing whether the stimulus was predominantly dark or light in four blocks of 104 trials each, totaling 416 responses, with speed and accuracy manipulations applied in certain blocks (for details, see Evans et al. (2017)). Data from six participants were excluded from the analysis based on their accuracy and RTs exceeding cutoff points (300–5000 ms).

**Table 3:** Summary statistics of posterior estimates from experimental data

	Models	$v_1$	$v_2$	*a*	*z*	$t_0$	$s_v$	$s_z$	$s_{t_0}$	$\alpha$
Speed	Basic DDM	1.30	$-$1.27	1.26	0.52	0.35				
		(0.45)	(0.49)	(0.22)	(0.06)	(0.07)				
	Basic LFM	1.27	$-$1.29	0.97	0.53	0.38				1.47
		(0.39)	(0.47)	(0.21)	(0.06)	(0.07)				(0.24)
	Full DDM	1.55	$-$1.53	1.04	0.53	0.44	0.45	0.13	0.22	
		(0.53)	(0.55)	(0.25)	(0.07)	(0.06)	(0.14)	(0.02)	(0.09)	
	Full LFM	1.31	$-$1.37	0.88	0.54	0.42	0.48	0.14	0.13	1.48
		(0.39)	(0.45)	(0.30)	(0.07)	(0.06)	(0.23)	(0.07)	(0.06)	(0.15)
Accuracy	Basic DDM	1.10	$-$0.97	1.96	0.53	0.43				
		(0.37)	(0.39)	(0.37)	(0.05)	(0.08)				
	Basic LFM	1.09	$-$0.95	1.82	0.53	0.45				1.85
		(0.36)	(0.41)	(0.40)	(0.06)	(0.08)				(0.10)
	Full DDM	1.42	$-$1.26	2.08	0.53	0.46	0.77	0.10	0.15	
		(0.38)	(0.43)	(0.88)	(0.06)	(0.08)	(0.21)	(0.06)	(0.08)	
	Full LFM	1.54	$-$1.41	2.14	0.54	0.46	0.99	0.12	0.10	1.78
		(0.33)	(0.45)	(0.56)	(0.06)	(0.08)	(0.30)	(0.02)	(0.08)	(0.13)

Notes: Summary statistics of all estimated parameters. *DDM*, diffusion decision model; *LFM*, Lévy-flight model; estimates correspond to grand means over $N= 43$ participants; parentheses denotes grand standard deviations

### Results

Table 3 summarizes the grand means and standard deviations of each parameter for all four models. As expected, results differed more strongly between DDM and LFM in the speed condition. Estimates of the $\alpha$ parameter were lower in the speed condition compared to the accuracy condition. In contrast, the parameter estimates from both models showed little to no discrepancy in the accuracy condition.

#### Basic Models

In the speed condition, mean estimates for $\alpha$ were approximately 1.5, suggesting that evidence accumulation deviates notably from a Wiener diffusion process. Boundary separation estimates were larger for the DDM compared to the LFM, which is consistent with the findings from our simulations, indicating that DDM results overestimate the boundary separation when $\alpha$ is small. As expected, the LFM estimated a larger non-decision time compared to the DDM. In the accuracy condition, $\alpha$ was closer to 2.0, indicating that the evidence accumulation process closely resembled the standard diffusion process. In this condition, both models provided similar estimates for boundary separation whereas estimates for non-decision time were still larger for the LFM.

#### Full Models

In the full models, inter-trial variability parameters were expected to account for possible deviations from a Wiener diffusion process. However, in speed conditions, estimates for boundary separation were still larger for the DDM compared to the LFM. The $\alpha$ parameter was estimated similarly to the basic model, indicating a continued deviation from the DDM process. In the accuracy condition, estimates for $\alpha$ were even smaller than in the basic models. While the LFM produced larger boundary separation estimates, other parameter estimates were nearly identical across models.

Overall, the effects of boundary separation and non-decision time in the speed-accuracy manipulation were captured in both model variants. Specifically, in the LFM, boundary separation was larger in the accuracy condition than in the speed condition, even though $\alpha$ indicated a deviation from the DDM process. Finally, non-decision time differs similarly across conditions in both models.

## Discussion

The DDM has been used for over four decades and is widely recommended to model behavior in fast, perceptual decisions (Ratcliff & McKoon, 2008). However, recent research suggests that a core assumption of the DDM may be violated in fast and easy tasks: for such tasks, evidence accumulation may be better described by a Lévy-Flight rather than by a Wiener diffusion process (Voss et al., 2019). Overlooking a Lévy-Flight pattern in evidence accumulation may not only reduce estimation precision but also introduce specific biases. This raises the question of whether findings from previously published diffusion model studies may include inaccurate interpretations due to subtle model misspecification. Previous results from LFM studies suggest this may be particularly relevant for studies comparing age groups or investigating speed-accuracy trade-offs (Voss et al., 2019; Wieschen et al., 2023). The aim of the present study was to determine if, and to what extent, DDM results are distorted when evidence accumulation follows a Lévy-Flight process.

We first established a baseline for parameter recovery by evaluating estimation quality of data simulated from the well-specified models. These results indicated reliable parameter estimation, regardless of model type (DDM or LFM), or estimation method (neural networks or MCMC). However, recovery was generally poor for the inter-trial variability parameters, a finding consistent with previous research (Lerche & Voss, 2016; Boehm et al., 2018) except for the findings by Henrich et al. (2024).

Our most important findings came from cross-fitting, where data simulated by the LFM was analyzed using the DDM. Note that the reverse (i.e., simulating with the DDM and recovering with the LFM) is unproblematic, as the DDM is a special case of the LFM. As expected, parameter recovery deteriorated for misspecified models. Specifically, our simulation results suggest that ignoring a Lévy-Flight pattern in evidence accumulation can lead to underestimation of non-decision-times and overestimation of boundary separations.

We then applied the models to experimental data from Evans et al. (2017), where a speed-accuracy manipulation was implemented. Estimates of the model parameters indicate the well-known effects of reduced boundary separation and non-decision time after speed instructions. Additionally, the LFM analyses revealed a reduction of $\alpha$ in this condition, indicating a deviation of evidence accumulation from a Wiener process. As in the simulation study, ignoring the reduced $\alpha$ resulted in an overestimation of boundary separation by the DDM results, and, consequently effects of instruction on boundaries were larger in the LFM (Table  3). Overall, estimates from both models exhibited similar patterns regarding how speed-accuracy instructions influenced the key model parameters.

### Implications for Previous DDM Studies

As sketched above, simulation results may indicate implications for previously published studies using speed-accuracy manipulations. If we assume that under accuracy-focused conditions, evidence accumulation is well described by a Wiener diffusion process, whereas it shifts to a Lévy-Flight under speed-focused conditions, parameters could be biased in the speed condition in such a way that drift rates and boundary separations are overestimated, non-decision times are underestimated. To elaborate, for the same fast response, the DDM is likely to infer a larger boundary and a higher drift to account for reaching the decision boundary quickly, while estimating a very short non-decision time. In contrast, the LFM assumes a narrower boundary and a lower drift because lower $\alpha$ values in fast responses introduce larger variance in the accumulation process, enabling the boundary to be reached without requiring an unrealistically short non-decision time. Consequently, the LFM might estimate longer non-decision time while estimating a narrower boundary and lower drift when $\alpha$ is low.

A similar implication may hold for studies on cognitive aging, where initial findings suggest that younger participants’ decision-making might be characterized by the LFM, while older participants’ performance is well explained by the standard DDM (Wieschen et al., 2023). Our simulations indicate that this discrepancy could overestimate boundary separation and drift rates, while underestimating non-decision times for younger participants. If the LFM indeed provided a better fit for younger individuals, it would suggest narrower boundary separation, lower drift rates, and longer non-decision times compared to DDM-based estimates. This is because lower $\alpha$ values allow the decision boundary to be reached more quickly without requiring extremely short non-decision times. Future studies could further investigate these effects through experimental manipulations designed to directly assess the influence on $\alpha$ on age-related differences in decision-making.

Our simulation study provided insight into extreme cases that are not typically observed in current experimental settings, offering valuable directions for future research. While simulations can help illustrate theoretical distinctions between models, experimental data is needed to underscore the practical implications of potential model misspecification. Accordingly, our analysis of experimental data confirmed that drift rates are influenced by speed-accuracy instructions when allowed to vary freely (e.g., Rae et al., 2014; Lerche and Voss, 2018). In the basic models, drift rates decreased under accuracy condition, whereas the full models produced inconclusive results. Similarly, the age trend for drift rates remains inconsistent (von Krause et al., 2022), with some studies even reporting higher drift rates for older adults in verbal tasks. In such cases, it is possible that lower drift rates for younger participants could have been compensated by lower $\alpha$ parameters. Additionally, well-established age-related effects on drift rates and boundaries—where robust differences have often been reported (Theisen et al., 2021)—are likely to remain valid despite potential model-specific biases. More importantly, both model variants reached the same conclusions regarding speed-accuracy manipulations, aligning with findings by Dutilh et al. (2019b), who demonstrated that different research teams using different variants of decision-making models and inference techniques reaching a consensus over experimental manipulations.

### Lévy Versus Wiener

This paper is based on the assumption that evidence accumulation in fast binary decision-making is—at least for some tasks and groups—better described by a Lévy-Flight than by a diffusion process. The LFM allows to quantify the level of divergence from the assumptions of the DDM, as the stability of the noise distribution can be modeled as an additional free parameter. To explore this, our cross-fitting exercise examined the relationship between $\alpha$ and the DDM parameters across a range of $\alpha$ values. We observed that as $\alpha$ decreased in LFM-generated data—that is, noise distribution deviated more from Gaussian distribution—the divergence between key parameter estimates in the DDM and the true parameters in the LFM grew more pronounced.

In the empirical application, in the speed condition, $\alpha$ values ranged from as low as 1.18 to as high as 1.77 (basic LFM), highlighting the inter-individual variability in evidence accumulation even under identical task instructions. In contrast, under accuracy-focused conditions, $\alpha$ values were more constrained, ranging between 1.57 and 1.99 (see Appendix, Table 6). These findings suggest that $\alpha$ captures speed-accuracy instructions and provides additional information beyond traditional decision parameters, capturing meaningful differences in cognitive processing across individuals and conditions. Additionally, Rasanan et al. (2024) demonstrated an increasing trend in $\alpha$ as a function of task experience, suggesting that $\alpha$ may reflect a mixture of decision strategies. Moreover, previous research (e.g., Wieschen et al., 2020; 2023) has shown that the LFM captures group- and condition-specific differences in $\alpha$, raising the question of whether individuals may have distinct evidence accumulation processes.

The present results—as well as previous studies showing superior fit of the LFM compared to the DDM—raise the question which neural processes might be responsible for heavy-tailed noise in evidence accumulation. According to the central limit theorem, normally distributed noise is expected when it arises as a composite of multiple independent and identically distributed (iid) sources with finite variance. However, if these multiple random variables are strongly correlated or follow heavy-tailed distributions themselves, their combined effect may result in a heavy-tailed rather than Gaussian distribution. We argue that both conditions may apply to neural computation: on the one hand, ensembles of neurons often transmit activation simultaneously, introducing highly correlated fluctuations. On the other hand, while neural activity may exhibit low-level background noise on average, occasional large spikes (sudden bursts of neural activation) could be responsible for leptokurtic noise distributions in evidence accumulation (e.g., Poisson noise provides a good approximation at this level; Wang, 2002). In cases of a more careful processing after accuracy instructions, neural systems may filter or suppress incoming signals, as this kind of processing requires a more thorough accumulating of evidence, rather than reacting to the first available information. Such filtering mechanisms (e.g., temporal integration or synaptic inhibition) could reduce the kurtosis of the noise distribution, shifting it toward a more Gaussian-like form. Early work by Wang and colleagues (Wang, 2002; Lo & Wang, 2006) pointed out that the significant difference in time scales between the activity of individual neurons and the broader decision-making process remains an open question. Nonetheless, aggregate properties of low-level models with non-Gaussian noise can often be well approximated by diffusion models that assume Gaussian noise during evidence accumulation (Smith, 2010; Smith & McKenzie, 2011).

### Limitations and Open Questions

We would like to emphasize here that the biases observed in this study are substantial only if the misspecification is also significant, that is, if the stability parameter $\alpha$ is notably below 2.0; slight deviations from the DDM’s assumptions will lead only to minor biases, as they agree on the manipulations being made in the data. A second limitation of this study is the reliance on only two recent estimation procedures. MCMC estimates may be less precise compared to neural estimates due to the use of smaller datasets, a consequence of the computational limitations of MCMC methods. Neural networks, by contrast, can process larger datasets, which allows them to converge to more accurate estimates in this simulation study. However, further analysis is required to evaluate how other parameter estimation algorithms, such as fast-dm (Voss & Voss, 2007) or $\chi ^2$-based methods (Ratcliff & Tuerlinckx, 2002), which have been used for the majority of older DDM studies behave when analyzing Lévy-Flight data.

Another problem is that the LFM does not have a closed-form likelihood function, which prevents the use of traditional inference methods, necessitating the reliance on simulation-based methods (e.g., neural posterior estimation). Although these methods are becoming more accessible, they can be challenging for novice users. Additionally, they require generating large datasets for the training of the neural networks, which demand substantial computational resources and powerful GPUs.

Moreover, with the incorporation of the $\alpha$ parameter, the LFM shows a better capacity to fit data compared to the DDM. However, this opens up important questions regarding the psychological and theoretical interpretations of the $\alpha$ parameter. Further research should examine the psychological meaning of $\alpha$ using a large number of datasets in order to identify the specific conditions and factors that influence $\alpha$ values (e.g., Rasanan et al., 2024). Given that $\alpha$ influences within-trial processing, targeted experimental manipulations designed to modulate within-trial processing should be explored, and ideally combined with neuroimaging measures, as has been done in studies employing the DDM (e.g., Ghaderi-Kangavari et al., 2023)

Additionally, the LFM should be systematically compared against other prominent decision-making models—beginning with the DDM as present study—and extending to models such as the Linear Ballistic Accumulator (LBA; Brown and Heathcote, 2008; Donkin et al., 2011), and the Ornstein-Uhlenbeck Model (OUM; Busemeyer and Townsend, 1993; Heath, 2000) in terms of goodness-of-fit and parameter recovery. Such comparisons could clarify where to place the LFM within prominent decision-making models and how $\alpha$ maps onto main model parameters.

Lastly, an important open question remains: why should one question the assumption of Gaussian noise in the first place especially where there are strong grounds (e.g., Smith et al., 2014)? Future research should evaluate why the heavy-tailed distribution should be preferred over numerous alternative distributions.

### Conclusions

Taken together, this extensive cross-fitting simulation study demonstrates that significant biases may arise in parameter estimates in extreme cases. However, when applied to experimental data, models converged on the same conclusions, highlighting the robustness of speed-accuracy tradeoff manipulation. Nevertheless, the additional information introduced by $\alpha$ cannot be overlooked, as it raises new questions about the underlying cognitive processes. Consequently, we advocate for the consideration of the LFM in behavioral data analysis and further investigation into conditions under which $\alpha$ differs.

## Figures and Tables

**Fig. 4:**
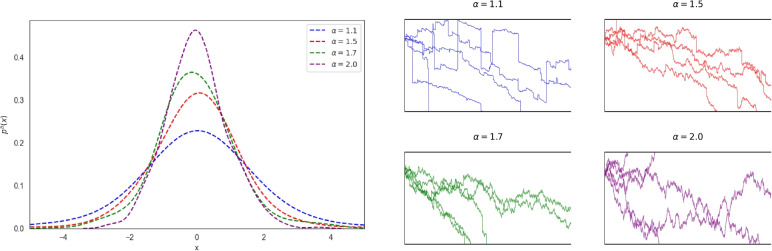
Distributions and accumulation processes across different $\alpha$ values. The left panel illustrates the noise distributions of the LFM for various $\alpha$ values, while the right panels display corresponding sample accumulation paths. When $\alpha = 2$, the noise follows a Gaussian distribution, making the model identical to the basic DDM. As $\alpha$ decreases below 2, the noise becomes increasingly heavy-tailed, leading to greater variability and more frequent large jumps in the accumulation process, visible as sudden spikes in the paths

**Fig. 5:**
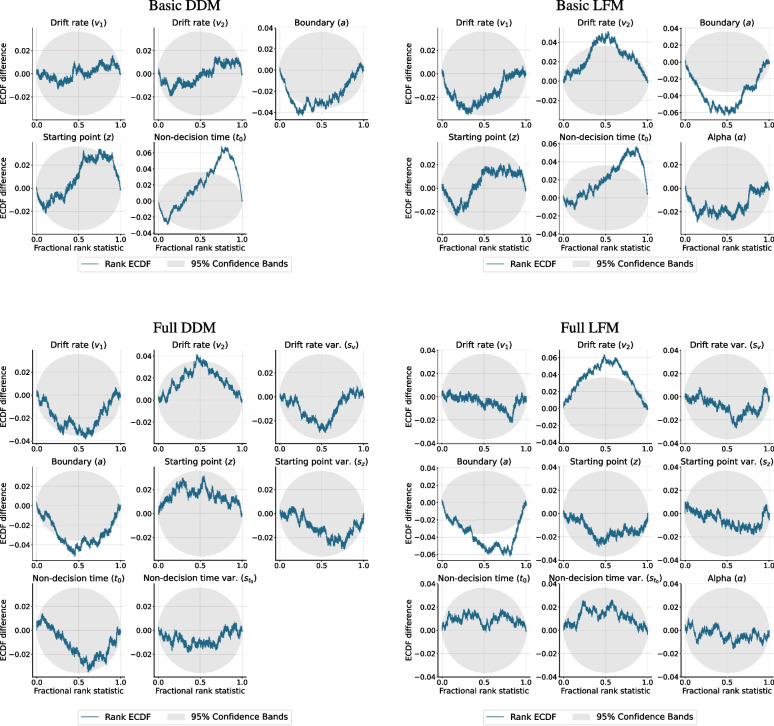
ECDF difference plots for basic and full models. The top row compares the basic DDM and LFM (Top panel), while the bottom row compares the full DDM and LFM (Bottom panel)

**Fig. 6:**
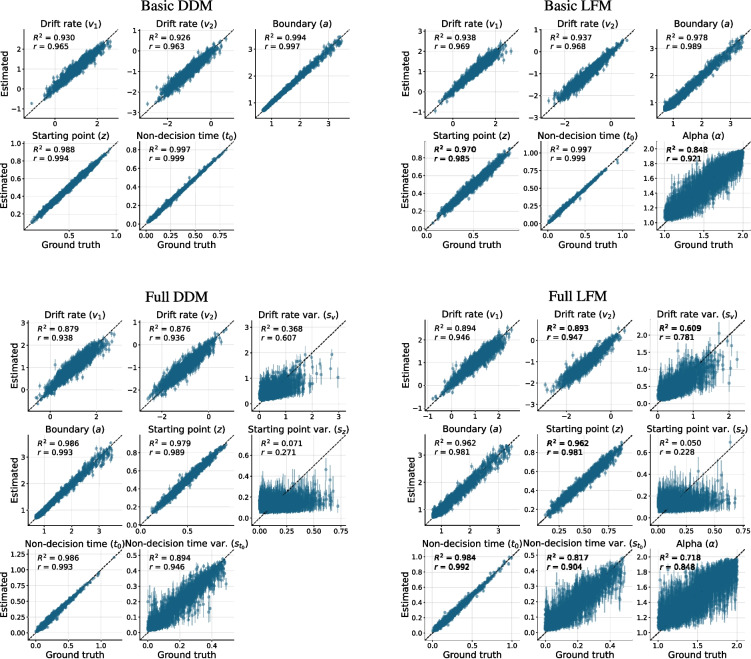
Parameter recovery plots for well-specified models. The top row illustrates the parameter recovery performance of the basic DDM and LFMs, while the bottom row illustrates the parameter recovery performance of the full DDM and LFMs

**Fig. 7:**
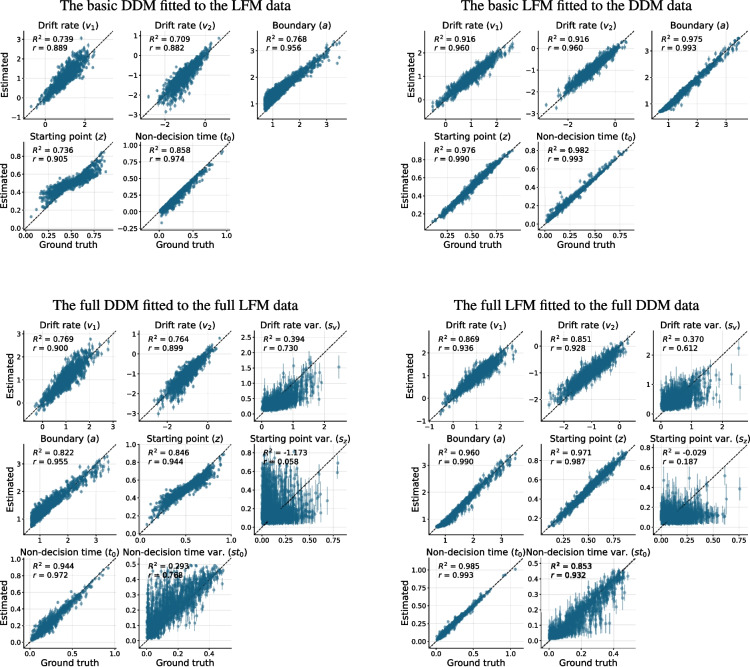
Parameter recovery plots for cross-fits of basic and full models. The top section illustrates the performance of basic models, while the bottom section illustrates the performance of full models

## Data Availability

No datasets were generated or analysed during the current study.

## Appendix A: Data Simulation

Table 4 shows the priors for all model parameters used for simulations.

**Table 4:** Prior distributions for model parameters

Parameter	Prior distribution
$v_1$	$\mathcal {N}(1,.5)$
$v_2$	$\mathcal {N}(-1,.5)$
*a*	$\mathcal {G}(2,\frac{1}{2}) \text {T}[0.7, 3.5]$
*z*	$\mathcal {B}(5, 5)$
$t_0$	$\mathcal {G}(3, \frac{1}{12})$
$s_v$	$\mathcal {G}(2, \frac{1}{4})$
$s_z$	$\mathcal {B}(1, 6)$
$s_{t_0}$	$\frac{\mathcal {B}(1, 2)}{2}$
$\alpha$	$\mathcal {U}(1, 2)$

Notes: $\mathcal {N}(a, b)$: normal distribution with mean *a* and standard deviation *b*; $\mathcal {B}(a, b)$: Beta distribution with shape parameters *a* and *b*; $\mathcal {G}(a, b)$: Gamma distribution with shape *a* and scale *b*; $\mathcal {U}(a, b)$: uniform distribution with a lower limit *a* and an upper limit *b*

From the priors presented above, we draw 1000 sets, from which we simulated datasets consisting of 500 trials for the basic and full versions of the DDM and the LFMs. For half of the trials of each data-set, drift $v_1$ and $v_2$ were used, respectively. Additionally, the inter-trial variability parameters were implemented as follow:

A.1 $$\begin{aligned} T_{v_i}&\sim \mathcal {N}(v_i , s_v), \end{aligned}$$

A.2 $$\begin{aligned} T_z&\sim \mathcal {U}(z - s_z, z + s_z), \end{aligned}$$

A.3 $$\begin{aligned} T_{t_0}&\sim \mathcal {U}(t_0 - s_{t_0}, t_0 + s_{t_0}). \end{aligned}$$

Table 5 shows the minimum, maximum, mean, and median RTs for each model, along with the standard deviation of RTs. The values are grand means over $N= 1000$ simulated participants.

**Table 5:** Descriptives for simulated RTs

Model	Mean absolute RT	Median RT	Std deviation	Minimum RT	Maximum RT
Basic DDM	0.69	0.51	0.37	0.28	2.91
Basic LFM	0.75	0.59	0.42	0.26	3.24
Full DDM	0.65	0.49	0.37	0.21	2.84
Full LFM	0.76	0.59	0.46	0.2	3.74

Figure 4 displays the noise distributions for representative $\alpha$ values in the left panel and the corresponding evidence accumulation trajectories in the right panel.

## Appendix B: Simulation-based Calibration

We evaluate the computational faithfulness of our Bayesian inference algorithm through simulation-based calibration. This approach relies on the premise that an ensemble of posterior distributions should closely resemble the prior distribution. If this condition holds true, we can confidently assert that the chosen inference method yields unbiased posterior distributions. To accomplish this, we generate 1000 datasets using our models (basic DDM and LFM; full DDM and LFM), fit the model to each dataset individually, and acquire 500 posterior samples per simulation. Results from the calibration based calibration are shown in Fig. 5.

## Appendix C: Parameter Recovery

Accurately retrieving the true data-generating parameters is crucial in cognitive modeling. Parameter recovery plots offer a visual representation of the correspondence between mean posterior estimates and the true values, with perfect recovery depicted by points falling precisely along the unity diagonal line. In such instances, credible intervals tend to be narrow, signaling minimal bias across the estimates. Parameter recovery performance for the well-specified models can be seen in Fig. 6.

Figure 7 illustrates the parameter recovery for misspecified models. The top row shows performance for the basic models. The DDM struggled to accurately recover the boundary separation and non-decision time parameters. In contrast, the LFM, which encompasses the DDM, showed good parameter recovery when fitted to DDM data. This performance was close to the closed-world performance of the LFM, as shown in Table 2. The lower row of Fig. 7 illustrates the cross-fitting performance of the full models. Similar to the basic DDM, the full DDM struggled to accurately recover the boundary separation and non-decision time parameters. Additionally, the recovery of inter-trial variability parameters was particularly poor. As with the basic LFM, the full LFM was able to include and account for the DDM data, demonstrating good recovery performance. However, inter-trial variability parameters were still recovered poorly, consistent with the closed-world performance of the full models (Table 2).

Figure 8 shows parameter recovery for the MCMC approach. Panel (a) depicts parameter recovery in MCMC when the data from the DDM, (b) when the data from LFM. The lower row of Fig. 8 illustrates the comparison the comparison of estimates from MCMC and neural estimation when the model is well-specified (c) and misspecified (d) conditions, *x*-axis shows MCMC estimates, *y*-axis is neural estimation estimates.

## Appendix D: Application of Experimental Data

Table 6 summarizes the 95% credible intervals for all the parameters for $N= 43$ participants.

**Table 6:** 95% credible intervals for the posteriors of parameters estimated from experimental data

	Models	$v_1$	$v_2$	*a*	*z*	$t_0$	$s_v$	$s_z$	$s_{t_0}$	$\alpha$
Speed	Basic DDM	[1.68–0.92]	[$-$0.89–($-$2.08)]	[1.37–1.16]	[0.57–0.46]	[0.37–0.33]				
	Basic LFM	[1.67–0.89]	[$-$0.89–($-$1.94)]	[1.16–0.80]	[0.61–0.45]	[0.41–0.35]				[1.77–1.18]
	Full DDM	[2.09–1.03]	[$-$1.01—($-$2.08)]	[1.18–0.91]	[0.59–0.46]	[0.48–0.40]	[1.08–0.06]	[0.42–0.00]	[0.31–0.14]	
	Full LFM	[1.88–0.82]	[$-$0.88–($-$1.94)]	[1.14–0.60]	[0.63–0.45]	[0.48–0.38]	[1.01–0.10]	[0.44–0.00]	[0.28–0.01]	[1.89–1.07]
Accuracy	Basic DDM	[1.39–0.82]	[$-$0.70–($-$1.25)]	[2.15–1.79]	[0.58–0.48]	[0.47–0.38]				
	Basic LFM	[1.39–0.78]	[$-$0.67–($-$1.23)]	[2.05–1.58]	[0.59–0.47]	[0.50–0.39]				[1.99–1.57]
	Full DDM	[1.95–0.97]	[$-$0.86–($-$1.72)]	[2.42–1.82]	[0.59–0.48]	[0.52–0.40]	[1.41–0.20]	[0.32–0.00]	[0.29–0.04]	
	Full LFM	[2.07–1.06]	[$-$0.96–($-$1.93)]	[2.55–1.80]	[0.61–0.48]	[0.51–0.40]	[1.54–0.47]	[0.37–0.00]	[0.26–0.01]	[1.99–1.40]

Notes: 95% of credible intervals for all estimated cognitive parameters from experimental data over $N= 43$ participants. The upper section shows estimates from the speed condition, and the lower section shows estimates from the accuracy condition. DDM: Diffusion Decision Model; LFM: Lévy-Flight Model; brackets indicate ranges of parameter estimates

## Footnotes

1 Sometimes, the diffusion constant (or intra-trial variability of drift; $\eta$) is presented as an additional model parameter. Note that $\eta$ represents the scale of noise and thus serves as a scaling parameter, whereas $\alpha$ governs the shape of the noise distribution, not its scale. To ensure parameter identifiability, the scaling parameter $\eta$ is usually fixed to 1 or 0.1.
